# Human decidual macrophages and NK cells differentially express Toll-like receptors and display distinct cytokine profiles upon TLR stimulation

**DOI:** 10.3389/fmicb.2014.00316

**Published:** 2014-07-01

**Authors:** Marion Duriez, Héloïse Quillay, Yoann Madec, Hicham El Costa, Claude Cannou, Romain Marlin, Claire de Truchis, Mona Rahmati, Françoise Barré-Sinoussi, Marie-Thérèse Nugeyre, Elisabeth Menu

**Affiliations:** ^1^Unité de Régulation des Infections Rétrovirales, Institut Pasteur, Département de VirologieParis, France; ^2^Centre d’Immunologie et des Maladies Infectieuses, INSERM U1135, Sorbonne Universités, UPMC Univ Paris 06Paris, France; ^3^Cellule Pasteur, Université Paris Diderot, Sorbonne Paris CitéParis, France; ^4^Unité d’Epidémiologie des Maladies Emergentes, Institut PasteurParis, France; ^5^UMR-CNRS-5164-CIRID, Université Bordeaux 2Bordeaux, France; ^6^Gynecology-Obstetrics Service, A. Béclère Hospital, AP-HPClamart, France; ^7^Gynecology-Obstetrics Service, Pitié Salpêtrière Hospital AP-HPParis, France

**Keywords:** decidua, TLR, NK cells, macrophages, innate mucosal immunity, cytokines, maternofetal interface

## Abstract

Maternofetal pathogen transmission is partially controlled at the level of the maternal uterine mucosa at the fetal implantation site (the decidua basalis), where maternal and fetal cells are in close contact. Toll-like receptors (TLRs) may play an important role in initiating rapid immune responses against pathogens in the decidua basalis, however the tolerant microenvironment should be preserved in order to allow fetal development. Here we investigated the expression and functionality of TLRs expressed by decidual macrophages (dMs) and NK cells (dNKs), the major decidual immune cell populations. We report for the first time that both human dMs and dNK cells express mRNAs encoding TLRs 1-9, albeit with a higher expression level in dMs. TLR2, TLR3, and TLR4 protein expression checked by flow cytometry was positive for both dMs and dNK cells. *In vitro* treatment of primary dMs and dNK cells with specific TLR2, TLR3, TLR4, TLR7/8, and TLR9 agonists enhanced their secretion of pro- and anti-inflammatory cytokines, as well as cytokines and chemokines involved in immune cell crosstalk. Only dNK cells released IFN-γ, whereas only dMs released IL-1β, IL-10, and IL-12. TLR9 activation of dMs resulted in a distinct pattern of cytokine expression compared to the other TLRs. The cytokine profiles expressed by dMs and dNK cells upon TLR activation are compatible with maintenance of the fetotolerant immune environment during initiation of immune responses to pathogens at the maternofetal interface.

## Introduction

The maternofetal interface is a unique anatomical site, in that it maintains a state of local immune tolerance to the semiallogenic fetus while simultaneously ensuring host defenses against microbial pathogens. Intrauterine bacterial and viral infections are considered to be associated to pregnancy disorders (preterm labor, fetal growth restriction, stillbirth) (Goldenberg and Thompson, 2003; Adams Waldorf and McAdams, 2013). Bacteria are able to shuttle from the lower genital tract to the non-pregnant intrauterine cavity (Egli and Newton, 1961) and are detected at the maternofetal interface (DiGiulio et al., 2008; Han et al., 2009; Castro-Leyva et al., 2012). Viruses are also present at the maternofetal interface (Chouquet et al., 1997; Fidler et al., 2004; Picone et al., 2013). Among them, herpes simplex virus, human immunodeficiency type 1 virus and human cytomegalovirus can infect placental cells (Douglas et al., 1991; Norskov-Lauritsen et al., 1992; Lagaye et al., 2001; Weisblum et al., 2011). However, *in utero* transmission of these viruses is relatively rare and seems to be controlled (Chouquet et al., 1997; Fidler et al., 2004; Picone et al., 2013). To ensure host defenses against invading pathogens, the maternofetal interface must efficiently recognize a broad range of pathogen-associated molecular patterns (PAMPs) in order to provide an immediate immune response.

The maternofetal interface is composed of the placenta (of fetal origin) and the maternal uterine mucosa (decidua) (Moffett-King, 2002). The decidua basalis is located at the implantation site, in close contact with the placenta and the maternal blood. Up to 40% of all decidua basalis cells are leukocytes. During the first trimester of pregnancy, NK cells (dNKs) account for 70% of decidual leukocytes, T cells for 10%, and CD14^+^ antigen-presenting cells for 20% (Trundley and Moffett, 2004; Houser et al., 2011; Svensson et al., 2011). CD14^+^ antigen-presenting cells display a macrophage-like phenotype and are thus referred to here as decidual macrophages (dM) (Trundley and Moffett, 2004; Houser et al., 2011; Svensson et al., 2011). Decidual immune cells have to maintain a tolerant environment and thus play a crucial role in embryo implantation and fetal development. DMs promote fetal implantation by secreting soluble factors and are also involved in tissue remodeling (Houser et al., 2011). Decidual NK cells are involved in angiogenesis and spiral artery remodeling, and regulate decidual invasion by placental trophoblast cells (Hanna et al., 2006; Lash et al., 2006a,b). Besides these crucial functions of inducing and maintaining a tolerant microenvironment, dMs and dNK cells might also have the critical task of initiating a rapid immune response against invading pathogens.

Toll-like receptors (TLRs) are innate immune receptors able to sense a broad variety of PAMPs, thereby contributing to frontline defenses against pathogens. Ten human TLR genes (TLR1-10) have been identified, encoding receptors with a leucine-rich repeat ectodomain that recognizes PAMPs (Guan et al., 2010; Kawai and Akira, 2011). TLR1, TLR6, and TLR10 form heterodimers with TLR2. Microbial membrane patterns are detected by cell-surface TLR1/2, TLR2, TLR4, TLR5, TLR2/6, and TLR2/10, while pathogen nucleic acid sequences are recognized by TLR3, TLR7, TLR8, and TLR9 located in intracellular vesicles (Guan et al., 2010; Kawai and Akira, 2011). PAMP recognition by TLRs induces the secretion of a large panel of cytokines, including pro-inflammatory cytokines (TNF-α, IL-1β, IL-6, and IL-8), type I/II interferons (IFN-α, IFN-β, IFN-γ), and chemokines, which in turn activate innate immune cells and direct adaptive immunity (Hart et al., 2005; Kwissa et al., 2012).

All TLR mRNAs are known to be expressed and to be modulated during the course of pregnancy (Canavan and Simhan, 2007; Krikun et al., 2007) in human total decidual cells that include maternal stromal and immune cells altogether with placental trophoblast cells which invade the mucosa. Moreover TLR2, TLR3, and TLR4 have been shown to be functional in total decidual tissue in the first trimester and/or at term (Canavan and Simhan, 2007; Krikun et al., 2007) and primary trophoblast cells are reported to have functional TLR2, TLR3, and TLR4 (Abrahams et al., 2004; Patni et al., 2009). To our best knowledge, there is so far no data about the expression and function of TLRs within the different immune cell subsets of the human decidua, particularly in dMs and dNK cells which are the more abundant innate immune cells in this mucosa.

The aims of this study were to investigate TLR expression in decidual macrophages and NK cells, and to characterize the cytokine profile resulting from TLR activation of both cell types, in order to understand the roles of these cells in antimicrobial defenses within a tolerogenic environment.

## Materials and methods

### Ethics statement

All the women donors in this study provided their written informed consent. The study was approved by Assistance Publique des Hôpitaux de Paris (n°VAL/2011/06-41/02), Agence de Biomédecine (n°PFS08-013) and the biomedical research committee of the Pasteur Institute, Paris, France (n°RBM/2005.024).

### Human decidua basalis tissue collection

Decidua basalis tissue samples were obtained from healthy women undergoing voluntary termination of pregnancy during the first trimester (8, 9, 10, 11, 12 weeks of gestation) at Antoine Béclère Hospital (Clamart, France) or Pitié-Salpêtrière Hospital (Paris, France).

### Isolation of decidual mononuclear cells, CD14^+^ decidual macrophages, and decidual NK cells

Decidual mononuclear cells were isolated as previously described (Marlin et al., 2012). Briefly, freshly isolated decidua basalis tissue was digested under agitation in PBS (Gibco)- 1 mg/ml collagenase IV (Sigma)- 50 U/ml DNase (Roche) for 1 h. Cell suspension was successively filtered through 100, 70, and 40 μm nylon cell strainer and mononuclear cells were isolated by a density gradient step with Lymphocyte Separation Medium (PAA). DMs and dNK cells were purified by flow sorting or by positive selection with magnetic beads. Cell sorting of both dM and dNK cells was performed on 2 decidua samples, decidual mononuclear cells were stained with Amcyan-labeled anti-CD45, Pacific Blue-labeled anti-CD14, Alexa700-labeled anti-CD56 (Becton Dickinson) and PE-TexasRed-labeled anti-CD3 (Beckman Coulter) antibodies. CD45^+^ CD14^+^ CD3^−^ CD56^−^ dMs and CD45^+^ CD14^−^ CD3^−^ CD56^bright^ dNK cells were sorted with a MoFlo ASTRIOS device (Beckman Coulter). DM and dNK cells flow sorted purity was >99%. DMs and dNK cells were also purified from decidual mononuclear cells by using anti-CD14^+^ and anti-CD56^+^ magnetic beads, respectively, as recommended by the manufacturer (Miltenyi). DM and dNK cell purity was controlled by flow cytometry with an LSRII 2-Blue 2-Violet 3-Red 5-Yelgr laser configuration (BD Biosciences) and FlowJo 9.1.3. software (Tristar). Magnetic bead purified dM and dNK cells purity was 90 ± 1.17 and 89.8 ± 2.38 respectively (mean ± s.e.m.). Contaminating cells were mainly CD45^−^ cells in both dM and dNK cell samples. DM and dNK cells were cultured in Ham F12: DMEM glutamax (V:V) medium (Gibco) supplemented with 15% of fetal calf serum (PAA), penicillin (0.1 U/l) and streptomycin (1.10^−8^ g/l) (Gibco).

### Real-time RT-PCR of TLR mRNAs in dMs and dNK cells

Total RNA was extracted from purified dM and dNK cells and from decidual mononuclear cells by using the RNeasy Mini kit (Qiagen) and on-column DNase digestion using the RNase-free DNase set (Qiagen). Total RNA (10 ng) was reverse transcribed with the High Capacity cDNA Reverse Transcription kit (Applied Biosystems), and genes of interest were pre-amplified with the Taqman PreAmp Master Mix as recommended by the manufacturer (Applied Biosystems). The resulting cDNA was used as a template for Taqman real-time PCR on an ABI 7700 Prism instrument (Applied Biosystems). Relative gene expression levels were determined with primer/probe sets from Applied Biosystems (Table 1). *CDKN1B* served as an endogenous control to normalize loaded cDNA samples. Relative gene expression between dM or dNK samples and a reference decidual mononuclear cell sample was determined by the 2^−ΔΔct^ method.

**Table 1:** **Applied Biosystems primer/probe sets used for realtime qPCR of TLR mRNAs**.

	**Primer/probe sets**
TLR1	Hs00413978_m1
TLR2	Hs01014511_m1
TLR3	Hs00152933_ml
TLR4	Hs00152939_m1
TLR5	Hs00152825_m1
TLR6	Hs01039989_s1
TLR7	Hs00152971_m1
TLR8	Hs00607866_mH
TLR9	Hs00370913_s1
CDKN1B	Hs00153277_m1

### Flow cytometry analysis of TLR2, TLR3, and TLR4 expression in dM and dNK cells

Immediately after isolation, decidual mononuclear cells were incubated with FcR blocking reagent (Miltenyi) and stained with Amcyan-labeled anti-CD45, Pacific Blue-labeled anti-CD14, Alexa700-labeled anti-CD56 (Becton Dickinson), PE-TexasRed-labeled anti-CD3 (Beckman Coulter) and APC-labeled anti-TLR2 or PE-labeled anti-TLR3 or APC-labeled anti-TLR4 (eBioscience) antibodies. For TLR3 staining, cells were permeabilized (Cytofix/Cytoperm kit, BD Biosciences). For each TLR staining, an isotype matched Ig was used as negative control. TLR expression on dMs (CD45^+^ CD14^+^ CD3^−^ CD56^−^) and dNK cells (CD45^+^ CD14^−^ CD3^−^ CD56^bright^) were analyzed by flow cytometry with an LSRII 2-Blue 2-Violet 3-Red 5-Yelgr laser configuration (BD Biosciences) and FlowJo 9.1.3. software (Tristar).

### dM and dNK cell stimulation with TLR agonists

Stimulation with TLR agonists were performed on dMs (*n* = 13) and dNK cell (*n* = 11) samples purified with magnetic beads. Purified dMs were seeded at 2 × 10^6^ cells/ml and stimulated or not for 24, 48 or 72 h with TLR agonists (Table 2) (Invivogen). Purified dNK cells were seeded at 2 × 10^6^ cells/ml with 2 ng/ml IL-15 and were stimulated for 24 or 72 h with TLR agonists, as for dMs. Supernatants were collected, cleared (5 min at 10 000 rpm) and stored at −80°C until cytokine quantification.

**Table 2:** **TLR agonists used for dM and dNK cell stimulation**.

	**Agonist**	**Concentration**
TLR2	HKLM	2.5 × 108 cells/ml
TLR3	Poly(I:C)	50 μg/ml
TLR4	LPS E. Coli K12	1 μg/ml
TLR7/8	R848	2.5 μg/ml
TLR9	Type C CpG oligonucleotide ODN M362	5 μM

### Multiplex cytokine analysis

Cytokines were measured by a Luminex assay with the Human Cytokine 25-plex antibody bead kit as recommended by the manufacturer (Invitrogen). For each sample, non-stimulated and stimulated conditions were run on the same Luminex plate.

### Statistical analysis and graphics

For each cytokine detected in a stimulated dM or dNK cell supernatant, we selected the time point corresponding to the largest fold change. A non-parametric permutation test for paired samples was used to compare the level of secretion in non-stimulated vs. stimulated conditions, *p* < 0.05 being considered significant. Statistical analyses were performed using R software (R Foundation for Statistical Computing, Vienna, Austria; http://www.R-project.org). Polar plots and histograms were generated with Sigmaplot version 12.5 and GraphPad Prism version 5.0.

## Results

### dMs and dNK cells express TLR1-9 transcripts

**Figure 1:**
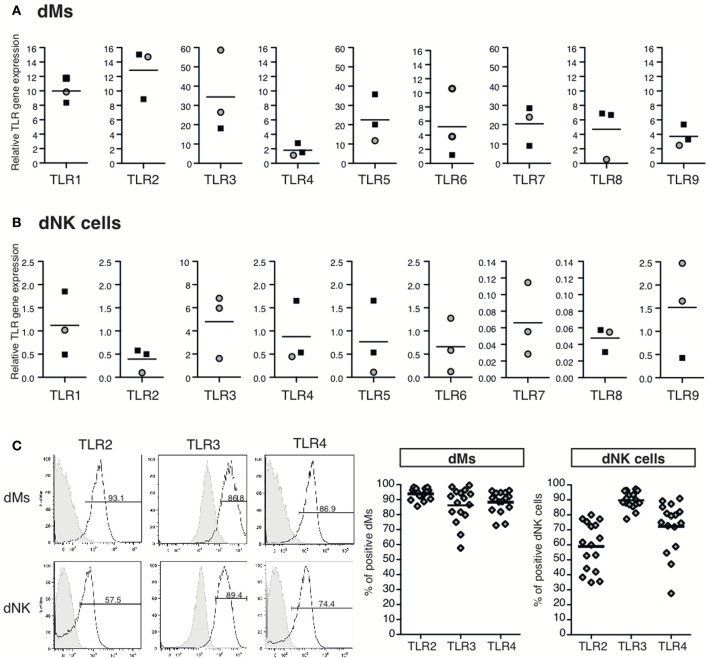
**Expression of TLR mRNAs and of TLR2, TLR3, and TLR4 protein by dM and dNK cells**. TLR1, TLR2, TLR3, TLR4, TLR5 TLR6, TLR7, TLR8, and TLR9 mRNA expression in purified dMs **(A)** and dNK cells **(B)** was quantified by RT-qPCR. Each dot represents one sample, dMs and dNK cells purified by flow sorting or by magnetic beads are depicted by black squares and gray circles respectively. Relative TLR gene expression was determined by the 2^−ΔΔCT^ method. TLR gene expression was normalized to the endogenous gene CDKN1B and a decidual mononuclear cell sample was used as reference. TLR2, TLR3, and TLR4 protein expression on dMs and dNK cells were investigated by flow cytometry **(C)**. Immediately after isolation, decidual mononuclear cells were incubated with FcR blocking reagent and stained with specific anti-TLR antibodies, dMs (CD45^+^ CD3^−^ CD56^−^ CD14^+^) and dNK cells (CD45^+^ CD14^−^ CD3^−^ CD56^bright^) were gated and analyzed for TLR expression. TLR2, TLR4, and intracellular TLR3 expression on dMs and dNK cells of a representative sample is displayed (left panel). Percent of positive dMs and dNK cells for each TLR are represented (right panel), each dot represents one sample (*n* = 16).

TLR expression by dMs and dNK cells has not previously been explored. For this purpose, reverse-transcribed RNA from a decidual mononuclear cell sample and from purified dMs and dNK cells were submitted to Taqman qPCR for TLR genes 1–9. As previously reported, all TLR mRNAs were detected in the decidual mononuclear cell sample (Canavan and Simhan, 2007; Krikun et al., 2007). In this sample, TLR4 mRNA was the strongest expressed transcript, whereas TLR3, TLR6, TLR7, TLR8, and TLR9 mRNA were less expressed (data not shown). The relative quantification of dM and dNK cell TLR transcripts was performed using this decidual mononuclear cell sample as reference. TLR1–9 transcripts were detected in dMs, with interindividual variability (Figure 1A). In dMs, TLR4, TLR6, TLR8, and TLR9 mRNA expression was lower than TLR1, TLR2, TLR5, and TLR7 mRNA expression, and TLR3 mRNA was the most strongly expressed (Figure 1A). All TLR mRNAs were also detected in dNK cells (Figure 1B) but at levels lower than in dMs and with interindividual variability. TLR7 and TLR8 mRNAs were weakly expressed in dNK cells, while TLR3 mRNAs was the most strongly expressed transcript (Figure 1B).

TLR2, TLR3, and TLR4 protein expression on dMs and dNK cells was further investigated by flow cytometry immediately after their isolation. The TLR expression on dMs and dNK cells of a representative sample is displayed on Figure 1C left panel. dMs and dNK cells expressed cell-surface TLR2 and TLR4 proteins, as well as intracellular TLR3. The percent medians of TLR expression on 16 decidual mononuclear cell samples are shown on Figure 1C right panel. TLR2, TLR3 and TLR4 proteins were respectively expressed on 93.89 ± 0.93, 86.12 ± 2.96, and 88.31 ± 1.82 (mean ± s.e.m.) percent of dMs. The percent of TLR2, TLR3, and TLR4 protein expressing dNK cells was lower than dMs. Indeed, 58.52 ± 4.05, 89.36 ± 1.41 and 71.96 ± 4.31 (mean ± s.e.m.) percent of dNK cells express TLR2, TLR3, and TLR4 protein. The interindividual variability of TLR2, TLR3, and TLR4 protein expression by dMs and dNK cells was not correlated to the term of pregnancy (data not shown).

Together, these results show that TLR1-9 mRNAs are expressed in both dMs and dNK cells, and that all TLR transcripts are expressed more strongly in dMs than in dNK cells. TLR2, TLR3, and TLR4 protein expression checked by flow cytometry was also positive on both dMs and dNK cells.

### dMs respond to TLR2, TLR3, TLR4, TLR7/8 and TLR9 agonists by enhancing their secretion of pro-inflammatory cytokines (IL-1β, TNF-α, IL-6, and IL-8)

**Figure 2:**
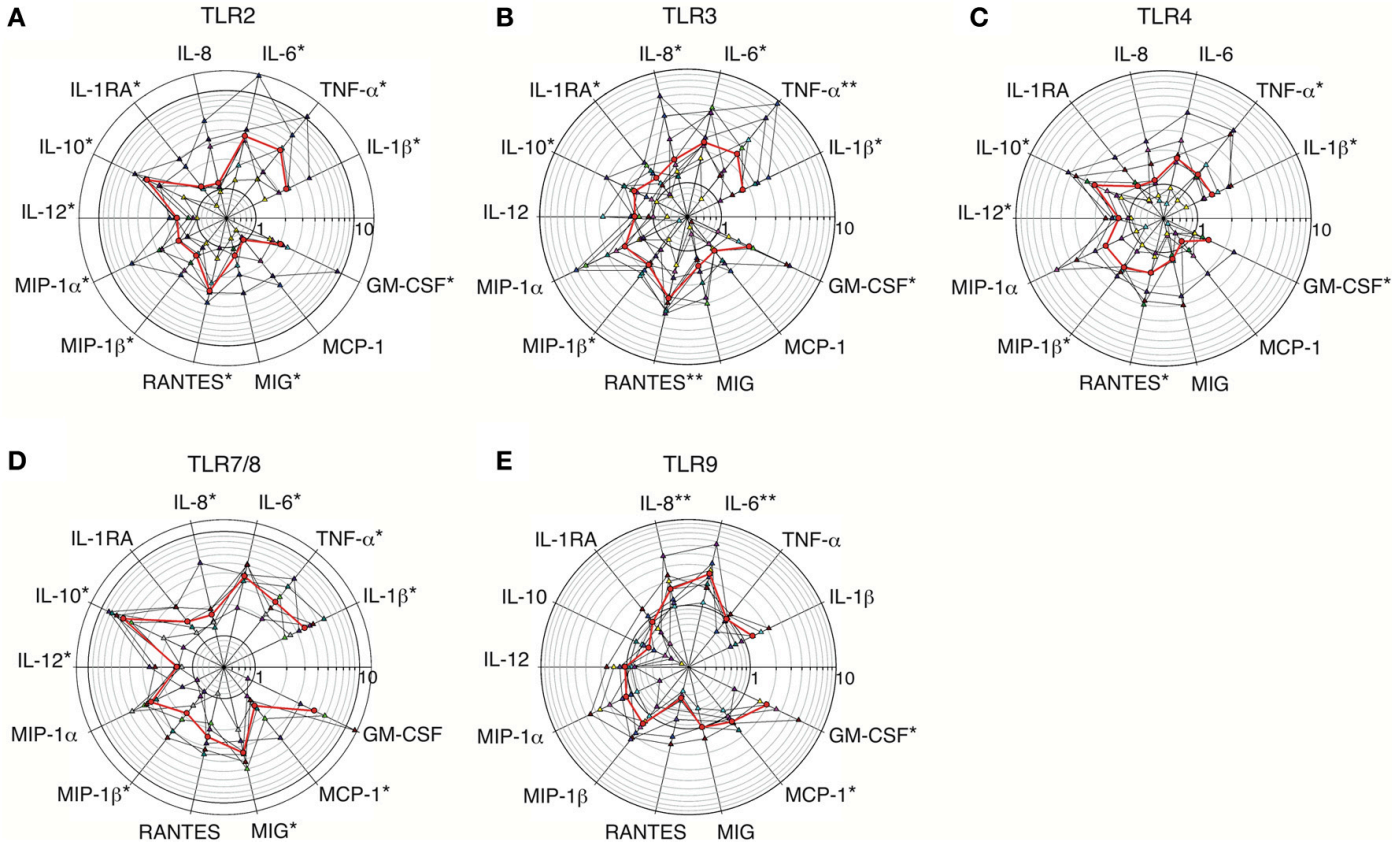
**Fold increases in cytokine secretion by dMs stimulated with TLR2, TLR3, TLR4, TLR7/8, and TLR9 agonists**. Fold increases in cytokine levels in supernatants of purified dMs incubated with TLR2 agonist (HKLM, *n* = 7) **(A)**; TLR3 agonist (poly(I:C), *n* = 9) **(B)**; TLR4 agonist (LPS, *n* = 7) **(C)**; TLR7/8 agonist (R848, *n* = 6) **(D)**, or TLR9 agonist (CpG ODN M362, *n* = 9) **(E)** are represented on polar plot with a common logarithmic scale (circles). Each colored triangle represents one sample and the red line represents the median. A non-parametric paired permutation was performed: ^*^p < 0.05 and ^**^p < 0.01.

To test TLR functionality, culture supernatants from primary dMs stimulated or not with specific agonists for 24–72 h were analyzed in a multiplex cytokine assay. Sixteen cytokines were detected in unstimulated dM supernatants (data not shown). Among them, 13 showed a statistically significant fold change in response to at least one TLR agonist (Figure 2 and Table 3). IL-2RA, IL-15, and IP-10/CXCL10 did not show any fold change in response to TLR agonists.

**Table 3:** **Cytokines modulated after TLR2, TLR3, TLR4, TLR7/8, and TLR9 stimulation of dMs**.

	**TLR2**			**TLR3**			**TLR4**			**TLR7/8**			**TLR9**		
	**Med. (Range) in pg/ml or in ng/ml^£^**		**Med. of F.I (IQR)**	**Med. (Range) in pg/ml or in ng/ml^£^**		**Med. of F.I (IQR)**	**Med. (Range) in pg/ml or in ng/ml^£^**		**Med. of F.I (IQR)**	**Med. (Range) in pg/ml or in ng/ml^£^**		**Med. of F.I (IQR)**	**Med. (Range) in pg/ml or in ng/ml^£^**		**Med. of F.I (IQR)**
	**US**	**S**		**US**	**S**		**US**	**S**		**US**	**S**		**US**	**S**	
IL-1β	142	250	2.39^*^	56	135	1.75^*^	108	133	1.50^*^	54	239	3.63^*^	56	113	1.29
	(45–595)	(193–1166)	(1.96–2.50)	(30–166)	(31–446)	(1.53–2.37)	(33–595)	(75–748)	(1.24–1.93)	(30–257)	(122–656)	(2.70–4.37)	(30–144)	(27–186)	(0.85–1.86)
TNF-α	230	934	3.83^*^	230	843	2.55^**^	230	459	1.55^*^	586	2037	3.12^*^	349	383	0.98
	(34–3315)	(54–1250)	(1.08–5.02)	(34–982)	(26–1029)	(1.50–3.66)	(9–3315)	(27–821)	(1.45–3.19)	(225–1857)	(438–167)	(2.31–4.53)	(48–720)	(30–1291)	(0.91–1.13)
IL-6	16.5^£^	40.9^£^	3.60^*^	2.0^£^	4.8^£^	2.36^*^	16.5^£^	20.9^£^	1.74	1.3^£^	5.8^£^	3.91^*^	2.7^£^	7.1^£^	2.48^**^
	(1.2–31.0)	(5.1–60.5)	(2.49–3.94)	(0.7–16.6)	(3.0–28.0)	(2.25–3.46)	(1.2– 31.0)	(2.4–30.0)	(1.27–2.19)	(0.7–6.0)	(1.3–26.9)	(3.56–4.80)	(0.7–31.0)	(2.5–45.1)	(1.86–2.79)
IL-8	464.9^£^	1323.2^£^	1.16	344.2^£^	564.1^£^	1.64^*^	448.0^£^	492.7^£^	1.10	42.8^£^	109.7^£^	1.63^*^	455.1^£^	553.9^£^	1.62^**^
	(25.4–1449.5)	(85.5–3204.6)	(1.03–3.06)	(30.7–1410.3)	(151.1–1857.7)	(1.38–3.30)	(30.7–1449.5)	(67.1–2374.7)	(0.91–1.81)	(19.6–572.6)	(27.2–697.5)	(1.39–1.86)	(19.6–1449.5)	(34.4–2850.5)	(1.13–2.02)
IL-1RA	1140	2397	1.28^*^	670	1058	1.36^*^	1140	2353	1.15	1051	1896	1.80	812	802	0.89
	(519–3069)	(376–3757)	(1.21–1.82)	(475–1234)	(422–2500)	(1.07–1.70)	(499–3069)	(419–2632)	(0.97–1.51)	(499–2629)	(1043–3455)	(1.54–2.71)	(475–3069)	(294–1292)	(0.69–1.03)
IL-10	989	5019	3.95^*^	492	705	1.64^*^	841	2454	2.35^*^	310	1819	5.86^*^	596	425	0.62
	(391–3625)	(899–13,424)	(2.86–4.79)	(167–1380)	(342–2150)	(1.22–2.06)	(316–3625)	(767–9478)	(1.61–3.19)	(167–1866)	(256–7021)	(2.84–7.52)	(191–1381)	(60–1230)	(0.37–0.79)
IL-12	36	68	1.59^*^	36	58	1.45	52	85	1.24^*^	47	94	1.41^*^	36	41	1.04
	(23–112)	(39–135)	(1.16–1.66)	(23–96)	(30–128)	(1.10–1.52)	(23–112)	(31–135)	(1.12–1.38)	(33–92)	(48–133)	(1.35–2.07)	(23–89)	(21–126)	(0.88–1.41)
MIP-1α	32.8^£^	70.7^£^	1.73^*^	12.0^£^	30.3^£^	2.03	32.8^£^	47.8^£^	1.83	13.6^£^	36.5^£^	2.96	12.0^£^	17.1^£^	1.21
	(8.6–111.5)	(23.2–192.9)	(1.54–2.78)	(4.5–50.4)	(9.2–79.7)	(1.42–3.02)	(4.5–138.3)	(16.0–177.8)	(1.10–2.93)	(5.8–38.8)	(8.9–143.0)	(1.69–3.46)	(4.5–50.4)	(5.2–54.7)	(1.06–1.98)
MIP-1β	12.1^£^	14.8^£^	1.55^*^	6.54^£^	9.97^£^	1.75^*^	12.11^£^	27.39^£^	1.80^*^	4.4^£^	9.5^£^	1.84^*^	4.94^£^	9.0^£^	1.34
	(4.9–40.5)	(8.7–625.9)	(1.34–2.18)	(1.7–20.3)	(2.7–35.5)	(1.40–2.02)	(1.9–40.5)	(3.8–48.0)	(1.47–1.98)	(1.7–12.1)	(2.6–15.4)	(1.23–2.84)	(1.8–20.3)	(1.7–30.6)	(1.03–1.84)
RANTES	217	556	2.89^*^	136	341	2.74^**^	217	340	1.56^*^	108	146	2.43	147	74	0.45
	(129–1003)	(387–1066)	(1.86–3.01)	(23–419)	(69–673)	(1.79–3.46)	(23–1003)	(64–1025)	(1.33–2.37)	(12–209)	(45–408)	(1.85–3.13)	(23–420)	(17–177)	(0.35–0.93)
MIG	60	100	1.24^*^	36	43	1.40	52	69	1.17	16	71.59	3.48^*^	44	43	1.00
	(27–156)	(36–164)	(1.14–1.76)	(6–125)	(18–90)	(1.18–1.98)	(19–156)	(30–168)	(0.90–1.87)	(7–60)	(23–124)	(2.84–4.11)	(17–125)	(15–74)	(0.72–1.00)
MCP-1	63.61^£^	87.97^£^	0.96	8.4^£^	10.2^£^	1.21	105.3^£^	78.9^£^	0.91	10.55^£^	23.85^£^	1.49^*^	44.1^£^	41.5^£^	1.24^*^
	(8.9–313.3)	(25.3–299.8)	(0.72–3.76)	(1.7–138.1)	(1.0–139.7)	(1.01–1.56)	(8.9–313.3)	(18.7–358.7)	(0.74–1.23)	(0.6–50.5)	(0.9–77.9)	(1.30–2.01)	(5.1–138.1)	(5.7–143.4)	(1.11–1.31)
GM-CSF	249	576	2.39^*^	57	97	2.02^*^	223	309	1.39^*^	35	144	4.60	72	136	1.96^*^
	(19–810)	(94–1250)	(1.96–2.50)	(18–809)	(56–1029)	(1.43–3.44)	(12–810)	(46–821)	(1.11–1.92)	(12–133)	(120–166)	(2.68–7.55)	(18–809)	(50–1291)	(1.75–2.64)

*Med., Median; F.I, fold increase; IQR, interquartile range; US, unstimulated; S, stimulated; £, concentration in ng/ml*.

* p < 0.05;

** *p < 0.01*.

Secretion of the pro-inflammatory cytokines IL-1β, TNF-α, IL-6, and/or IL-8 was increased in dM supernatants after TLR2, TLR3, TLR4, TLR7/8, and TLR9 stimulation with specific agonists. Regarding TLRs that sense microbial membrane patterns, pro-inflammatory cytokine induction was stronger after TLR2 than TLR4 stimulation, except for IL-8 which secretion was weakly enhanced in both conditions (Figures 2A,C). TLR2 stimulation induced a statistically significant increase in IL-1β, TNF-α, and IL-6 secretion by dMs (median 2.39, 3.83, and 3.60-fold, respectively; Figure 2A and Table 3). Induction of these four pro-inflammatory cytokines by TLR4 stimulation was observed but showed interindividual variability (Figure 2C). Only a significant induction of IL-1β and TNF-α secretion was detected after TLR4 stimulation (median 1.5-fold for both cytokines; Figure 2C and Table 3).

Regarding TLRs that sense pathogenic nucleic acids, dM stimulation with TLR3 agonist induced significant secretion of the four pro-inflammatory cytokines, with median fold increases of 1.75 for IL-1β, 1.64 for IL-8, 2.55 for TNF-α, and 2.36 for IL-6 (Figure 2B and Table 3). TLR7/8 stimulation strongly and significantly enhanced secretion of IL-1β, TNF-α, IL-6, and IL-8 in all the samples, with median fold increases of 3.63, 3.12, 3.91, and 1.64, respectively (Figure 2D and Table 3). Among all the TLRs, TLR7/8 stimulation of dMs resulted in the strongest increase in IL-1β and IL-6 secretion (Table 3). The TLR9 agonist enhanced IL-1β, IL-6, and IL-8 but not TNF-α secretion (median fold changes of 1.29, 2.48, 1.62, and 0.89 respectively) (Figure 2E). TLR9 stimulation led to a statistically significant increase in IL-6 and IL-8 secretion in all the samples (Figure 2E and Table 3).

These results show that TLR2, TLR3, TLR4, TLR7/8, and TLR9 are functional in dMs, as their activation with specific agonists induces statistically significant increases in the secretion of at least one pro-inflammatory cytokine (IL-1β, TNF-α, IL-6, and/or IL-8).

### TLR2, TLR3, TLR4, and TLR7/8 but not TLR9 agonists enhance dM secretion of the anti-inflammatory cytokines IL-1RA and IL-10

**Figure 3:**
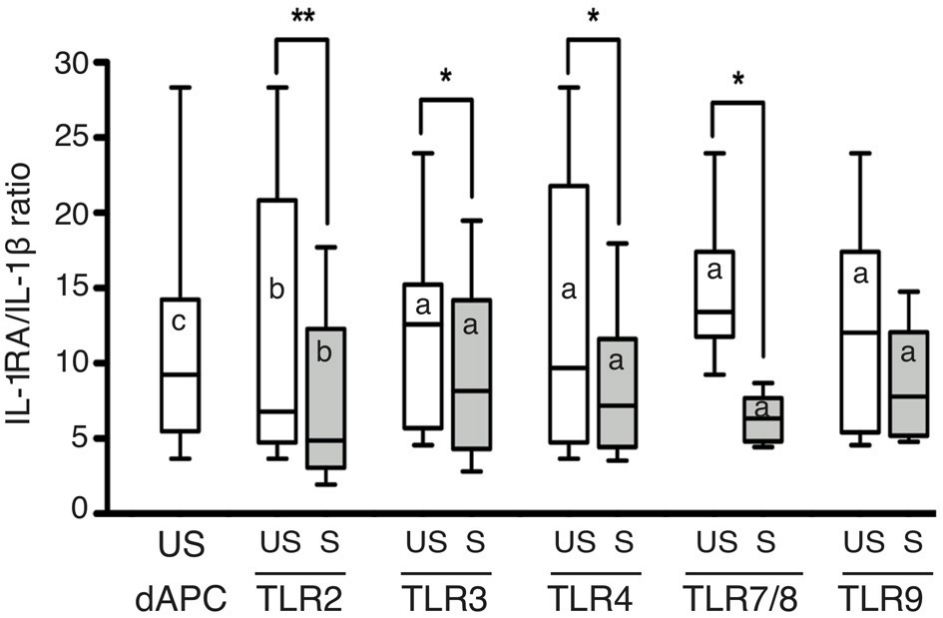
**Ratio of IL-1RA/IL-1 β secreted by CD14^+^ dMs after TLR2, TLR3, TLR4, TLR7/8, and TLR9 activation**. The IL-1RA/IL-1β ratio of unstimulated and stimulated dMs used for TLR2, TLR3, TLR4, TLR7/8, and TLR9 stimulation is reported (median and range). The IL-1RA/IL-1β ratio of all unstimulated (us) dM samples used for the study is also reported to the left of the graph (median and range). A non-parametric paired permutation was performed between IL-1RA and IL-1β secretion (^a^*p* < 0.05, ^b^*p* < 0.01, and ^c^*p* < 0.001) and between unstimulated and TLR-stimulated dMs IL-1RA/IL-1β ratio (^*^p < 0.05, ^**^p < 0.01).

Interestingly, dM activation by TLR agonists also enhanced the secretion of IL-1RA and IL-10, two anti-inflammatory cytokines. TLR2, TLR3, TLR4, and TLR7/8 activation led to a significant increase in IL-1RA secretion, with median fold increases of 1.15–1.80 after TLR2, TLR3, and TLR7/8 stimulation (Figures 2A–D). IL-1RA secretion was inhibited by TLR9 activation in 6 out of 8 test samples, with a median change of 0.89-fold (Figure 2E and Table 3). As IL-1RA antagonizes the pro-inflammatory cytokine IL-1β, and as the secretion of these two factors was generally enhanced 72 h post-stimulation, we compared their basal and stimulated secretion at this time point. Unstimulated dMs secreted significantly (10-fold) more IL-1RA than IL-1β (Figure 3). After TLR2, TLR3, TLR4 and TLR7/8 activation, the increase in IL1-β secretion reduced the IL-1RA/IL-1β ratio by 2.4- to 6.5-fold when compared to unstimulated conditions (Figure 3). However, the modest increase in IL-1RA secretion under TLR stimulation was enough to maintain this balance in favor of IL-1RA, as the median IL-1RA/IL-1β ratio was always higher than 5:1 (Figure 3). Interestingly, dM stimulation with TLR2, TLR3, TLR4, and TLR7/8 agonists led to a robust increase in IL-10 secretion (median fold increase 1.64–5.86) (Figures 2A–D). After TLR9 activation, IL-10 secretion by dMs declined in all samples (median 0.62-fold) (Figure 2E and Table 3).

Thus, TLR activation of dMs upregulated not only pro-inflammatory cytokines but also the anti-inflammatory cytokines IL-10 and IL1-RA. IL-10 was the strongest induced cytokine following TLR2, TLR4, and TLR7/8 activation. The pro and anti-inflammatory cytokine profile highlights a tolerogenic characteristic of dMs under TLR activation. One peculiar feature of dMs was the inhibition of IL-10 and IL-1RA secretion following TLR9 activation.

### TLR2, TLR3, TLR4, TLR7/8, and TLR9 activation of dMs modulates IL-12, β-chemokine, MIG, MCP-1, and GM-CSF secretion

Cytokines involved in immune cell crosstalk include IL-12, β-chemokines, MIG/CXCL9, MCP-1/CCL2, and GM-CSF. Unstimulated dMs secreted IL-12 (Table 3), and this secretion was upregulated by TLR2, TLR3, TLR4, and TLR7/8 agonists, by a median of 1.24- to 1.59-fold (Figures 2A–D and Table 3). IL-12 upregulation was significant for TLR2, TLR4, and TLR7/8 agonist stimulation (Figures 2A,C,D and Table 3).

The β-chemokines MIP-1α/CCL3, MIP-1β /CCL4, and RANTES/CCL5 were detected in the supernatants of unstimulated dMs (Table 3). MIP-1α secretion by dMs was upregulated by all the TLR stimulations, with a median of 1.21- to 2.96-fold (Figure 2 and Table 3). However, only MIP-1α enhancement for TLR2 activation was significant, owing to interindividual variability (Figure 2A). MIP-1β was significantly upregulated after TLR2, TLR4, TLR3, and TLR7/8 activation (Figures 2A–D; median fold increase respectively 1.55, 1.75, 1.80, and 1.84). RANTES secretion by dMs was enhanced by TLR2, TLR3, TLR4, and TLR7/8 agonists and was significant for TLR2, TLR3, and TLR4 (Figures 2A–D and Table 3). Interestingly, after TLR9 activation, RANTES secretion declined with a median of 0.45-fold (Figure 2E and Table 3).

DM secretion of MIG cytokine was also upregulated by TLR2, TLR3, TLR4, and TLR7/8 agonists, in a statistically significant manner for TLR2 and TLR7/8 (median fold increase 1.24 and 3.48, respectively; Figures 2A,D and Table 3). MCP-1 and GM-CSF are involved in monocyte/macrophage recruitment. MCP-1 secretion by dMs was enhanced after TLR3, TLR7/8, and TLR9 activation, and significantly for TLR7/8 and TLR9 (Figures 2B,D,E). GM-CSF secretion was enhanced by all the TLR agonists, significantly by TLR2, TLR3, TLR4, and TLR9 (Figure 2). TLR7/8 activation resulted in the largest increase in GM-CSF secretion (median fold increase 4.6), but the change did not reach statistical significance (Figure 2D and Table 3).

Together, these results show that dM secretion of IL-12 and GM-CSF and of several chemokines (MIP-1α, MIP-1β, RANTES, MIG, MCP-1) is enhanced by TLR activation.

### dNK cells respond to TLR2, TLR3, TLR4, TLR7/8, and TLR9 agonists by enhancing their secretion of pro-inflammatory cytokines (IFN-γ, IL-6 and IL-8)

**Figure 4:**
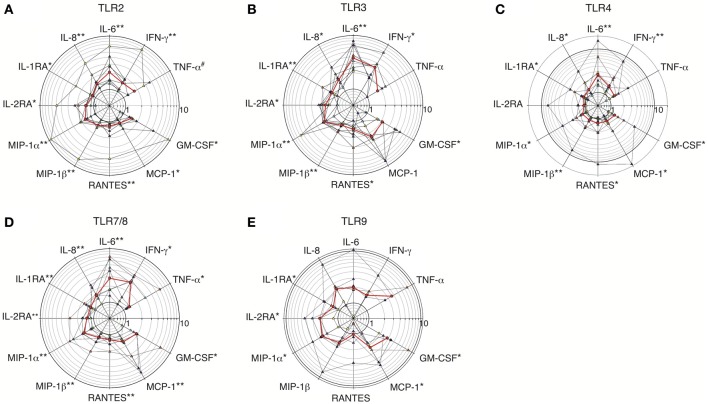
**Fold increases in cytokine secretion by dNK cells stimulated with TLR2, TLR3, TLR4, TLR7/8, and TLR9 agonists**. Fold increases in cytokine levels in the supernatants of purified dNK cells incubated with TLR2 agonist (HKLM, *n* = 8) **(A)**; TLR3 agonist (poly(I:C), *n* = 8) **(B)**; TLR4 agonist (LPS, *n* = 8) **(C)**; TLR7/8 agonist (R848, *n* = 8) **(D)** or TLR9 agonist (CpG ODN M362, *n* = 6) **(E)** are represen ted on polar plot with a common logarithmic scale (circles). Each colored triangle represents one sample, and the red line represents the median. A non-parametric paired permutation was performed: ^*^p < 0.05 and ^**^p < 0.01. ^#^Lowest probability that can be reached.

Culture supernatants of primary dNK cells incubated with TLR agonists for 24–72 h were analyzed as for dMs. Fourteen cytokines were spontaneously secreted by dNK cells (data not shown). Among them 11 showed a statistically significant fold change after TLR stimulation (Figure 4 and Table 4), whereas IL-12, IP-10, and MIG did not.

**Table 4:** **Cytokines modulated after TLR2, TLR3, TLR4, TLR7/8, and TLR9 stimulation of dNK cells**.

	**TLR2**			**TLR3**			**TLR4**			**TLR7/8**			**TLR9**		
	**Med. (Range) in pg/ml**		**Med. of F.I (IQR)**	**Med. (Range) in pg/ml**		**Med. of F.I (IQR)**	**Med. (Range) in pg/ml**		**Med. of F.I (IQR)**	**Med. (Range) in pg/ml**		**Med. of F.I (IQR)**	**Med. (Range) in pg/ml**		**Med. of F.I (IQR)**
	**US**	**S**		**US**	**S**		**US**	**S**		**US**	**S**		**US**	**S**	
TNF-α	37	45	1.72^#^	15	37	1.68	39	47	1.38	53	92	1.32^*^	15	118	3.61
	(8–222)	(21–315)	(1.26–2.73)	(8–82)	(8–172)	(1.00–2.09)	(8–222)	(14–228)	(1.08–1.69)	(8–88)	(9–252)	(1.20–2.51)	(8–82)	(28–208)	3.61
IFN-γ	5823	84	1.54^**^	39	84	3.27^**^	61	138	1.61^**^	43	60	3.00^*^	44	42	1.65
	(9–1343)	(25–2032)	(1.14–1.81)	(9–65)	(18–335)	(2.12–3.55)	(9–1343)	(66–2015)	(1.32–2.52)	(9–1343)	(34–4410)	(1.35–3.42)	(9–296)	(21–529)	(0.69–1.96)
IL-6	212	608	2.10^**^	75	503	3.80^*^	102	349	2.57^**^	58	285	2.80^**^	75	279	1.97
	(39–1770)	(110–2693)	(1.50–3.16)	(13–1392)	(57–3097)	(2.48–6.59)	(30–1342)	(60–3655)	(1.79–3.81)	(14–1392)	(19–2432)	(1.74–4.90)	(14–1392)	(31–2725)	(1.79–2.69)
IL-8	13,559	28,780	1.48^**^	10,308	23,907	1.76^**^	14,192	19,867	1.32^*^	10,308	16,960	1.57^**^	12,583	31,108	2.30
	(4198–49,318)	(9668–58,981)	(1.38–1.78)	(3097–66,424)	(5439–101,964)	(1.52–2.13)	(4198–66,424)	(5525–133,561)	(1.25–1.98)	(1267–112,494)	(1992–171,431)	(1.47–1.81)	(1267–152,559)	(2799–183,374)	(1.45–2.54)
IL-1RA	376	437	1.16^*^	222	319	1.44^*^	416	396	1.19^*^	263	380	1.29^**^	276	366	1.40^*^
	(107–811)	(217–902)	(1.12–1.30)	(107–424)	(186–465)	(1.19–1.53)	(107–811)	(221–1117)	(1.03–1.55)	(68–811)	(107–831)	(1.19–1.46)	(68–424)	(148–465)	(1.14–2.03)
IL-2RA	128	217	1.39^*^	248	464	1.66^**^	200	354	1.04	192	286	1.34^**^	109	298	2.23^*^
	(44–603)	(97–832)	(1.28–1.99)	(32–657)	(32–910)	(1.29–1.96)	(32–603)	(32–723)	(1.00–1.42)	(72–657)	(117–886)	(1.23–1.59)	(44–657)	(97–804)	(2.20–2.90)
MIP-1α	727	1525	1.66^**^	987	2094	2.12^**^	1115	1647	1.27^*^	845	1416	(248–12,185)	1.80^**^	605	1411
	(106–6489)	(367–9796)	(1.45–2.08)	(129–3329)	(291–20,723)	(2.04–2.26)	(106–3553)	(462–4377)	(1.04–1.98)	(128–6489)	(248-12,185)	(248–12,185)	(1.28–2.21)	(129–3068)	(523–3380)
MIP-1β	3477	3895	1.33^**^	2021	3431	1.36^**^	4472	4705	1.23^**^	2422	3774	1.30^**^	1800	2882	1.74
	(258–7984)	(1850–10,685)	(1.15–1.38)	(341–6092)	(604–7841)	(1.25–1.76)	(341–10,820)	(725–19,665)	(1.07–1.86)	(258–7984)	(418–13,031)	(1.20–1.62)	(258–5829)	(1895–4916)	(1.41–2.08)
RANTES	187	267	1.21^**^	291	420	1.41^*^	285	342	1.28^*^	373	493	1.27^**^	266	262	1.02
	(17–1576)	(83–2353)	(1.10–1.58)	(26–1576)	(45–1877)	(1.28–1.77)	(26–1576)	(120–2216)	(1.02–2.07)	(26–2906)	(40–3043)	(1.18–1.52)	(17–678)	(61–424)	(0.70–1.40)
MCP-1	787	748	1.19^*^	226	591	2.43	297	750	1.31^*^	203	594	1.61^**^	226	447	2.23^*^
	(187–3088)	(250–3525)	(1.10–1.29)	(37–11,388)	(97–16,104)	(1.34–4.09)	(37–4004)	(57–4497)	(1.11–1.75)	(64–8118)	(259–12,139)	(1.42–3.98)	(64–11,389)	(304–21,969)	(1.79–4.19)
GM-CSF	175	298	1.49^*^	134	226	2.14^*^	278	292	1.40^*^	177	292	1.89^*^	108	307	2.86^*^
	(34–3124)	(107–4250)	(1.25–2.54)	(25–741)	(74–1310)	(1.73–4.03)	(34–3124)	(117–4416)	(1.13–1.90)	(11–3124)	(67–4439)	(1.53–1.96)	(25–528)	(90–989)	(2.33–3.46)

*Med., Median; F.I, fold increase; IQR, interquartile range; US, unstimulated; S, stimulated*.

* p < 0.05;

** *p < 0.01*.

# *Lowest probability that can be reached*.

Unstimulated dNK cells secreted the pro-inflammatory cytokines IFN-γ, TNF-α, IL-6, and IL-8, and these four cytokines were upregulated after TLR2, TLR3, TLR4, TLR7/8, and TLR9 stimulation (Table 4). TNF-α was weakly expressed by dNK cells, and only by 8 of the 14 samples tested. Thus, although TNF-α secretion was enhanced by TLR3, TLR4, and TLR9 stimulation, the change was not statistically significant because of the small number of samples in which it was detected (Figure 4 and Table 4).

TLR2 stimulation significantly enhanced IFN-γ, IL-6, and IL-8 secretion by dNK cells (median fold increase 1.54, 2.10, and 1.48, respectively), and all the samples responded (Figure 4A and Table 4). TNF-α secretion was detected and enhanced in 5 of 8 samples submitted to TLR2 stimulation, and the *p*-value was the smallest possible given the sample size available (*p* = 0.062) (Table 4). IFN-γ, IL-6, and IL-8 secretion by dNK cells was also significantly enhanced upon TLR4 stimulation, with median fold increases of respectively 1.61, 2.57, and 1.32 (Figure 4C and Table 4). Regarding intracellular TLRs, TLR3, and TLR7/8 stimulation significantly upregulated IFN-γ, IL-6, and IL-8 secretion by dNK cells, by more than 3-fold for IFN-γ and IL-6 (Figures 4B,D and Table 4). TLR7/8 stimulation led to a significant increase in TNF-α secretion (median fold increase 1.32), TNF-α being detected and upregulated in 6 of the 8 samples tested (Figure 4D and Table 4). TLR9 stimulation also enhanced IFN-γ, IL-6, and IL-8 secretion by dNK cells (Figure 4E and Table 4), but the increase in these pro-inflammatory cytokines did not reach statistical significance, owing to interindividual variability.

These results show that TLR2, TLR3, TLR4, and TLR7/8 are functional in dNK cells, as their stimulation by specific agonists significantly upregulates the pro-inflammatory cytokines IFN-γ, IL-6, and IL-8. The response to TLR9 stimulation was highly variable: the pro-inflammatory cytokines were upregulated in some samples, but none of the median fold increases was statistically significant.

### TLR2, TLR3, TLR4, TLR7/8, and TLR9 activation modulates dNK cell secretion of anti-inflammatory cytokines, β-chemokines, MCP-1 and GM-CSF

IL-1RA and IL-2RA were the only anti-inflammatory cytokines secreted by dNK cells. Basal IL-1RA secretion was 3-fold lower than that observed with dMs (data not shown). IL-1RA secretion by dNK cells was significantly enhanced by all the TLR agonists (Figure 4). As CD56^bright^ CD16^−^ NK cells are reported to constitutively express the high-affinity IL-2 receptor (IL2RA/IL2RB/IL2RG) (Robertson, 2002), it was not surprising to observe IL-2RA secretion by unstimulated dNK cells. IL-2RA secretion was significantly upregulated by TLR2, TLR3, TLR7/8, and TLR9 stimulation, whereas TLR4 activation did not reproducibly increase IL-2RA secretion (Figure 4C).

The β-chemokines MIP-1α, MIP-1β, and RANTES were detected in dNK cell supernatants at basal levels respectively 16-fold and 3-fold lower than and identical to those of dMs (data not shown). DNK cells activated by TLR2, TLR3, TLR4, and TLR7/8 agonists significantly upregulated their secretion of the three β-chemokines, by a median of 1.27- to 2.12-fold for MIP-1α, 1.23- to 1.36-fold for MIP-1β, and 1.21- to 1.41-fold for RANTES (Figures 4A–D and Table 4). TLR9 activation significantly enhanced MIP-1α secretion (median 2.46-fold); MIP-1β secretion was also enhanced but the change did not reach statistical significance (Figure 4E). Half of the 6 tested dNK cell samples showed an increase or decrease in RANTES secretion after TLR9 activation (Figure 4E).

DNK cells secreted both MCP-1 and GM-CSF. The basal level of MCP-1 secretion was 30-fold lower than that observed with dMs, while GM-CSF secretion was of the same order with the two cell types (data not shown). Significant increases in MCP-1 secretion by dNK cells were observed after TLR2, TLR4, TLR7/8, and TLR9 activation (Figures 4A,C–E). TLR3 activation induced the largest increase in MCP-1 secretion (median 2.43-fold), but the change was not statistically significant because secretion was inhibited in one sample (Figure 4B and Table 4). GM-CSF secretion after TLR2, TLR3, TLR4, TRL7/8, and TLR9 activation was significantly increased (Figure 4).

Together, these results show that TLR activation enhances dNK cell secretion of the anti-inflammatory cytokines IL-1RA and IL-2RA, the chemokines MIP-1α, MIP-1β, RANTES, and MCP-1, and the cytokine GM-CSF.

### Comparison of cytokine profiles of dMs and dNK cells activated by intracellular and cell-surface TLRs

**Figure 5:**
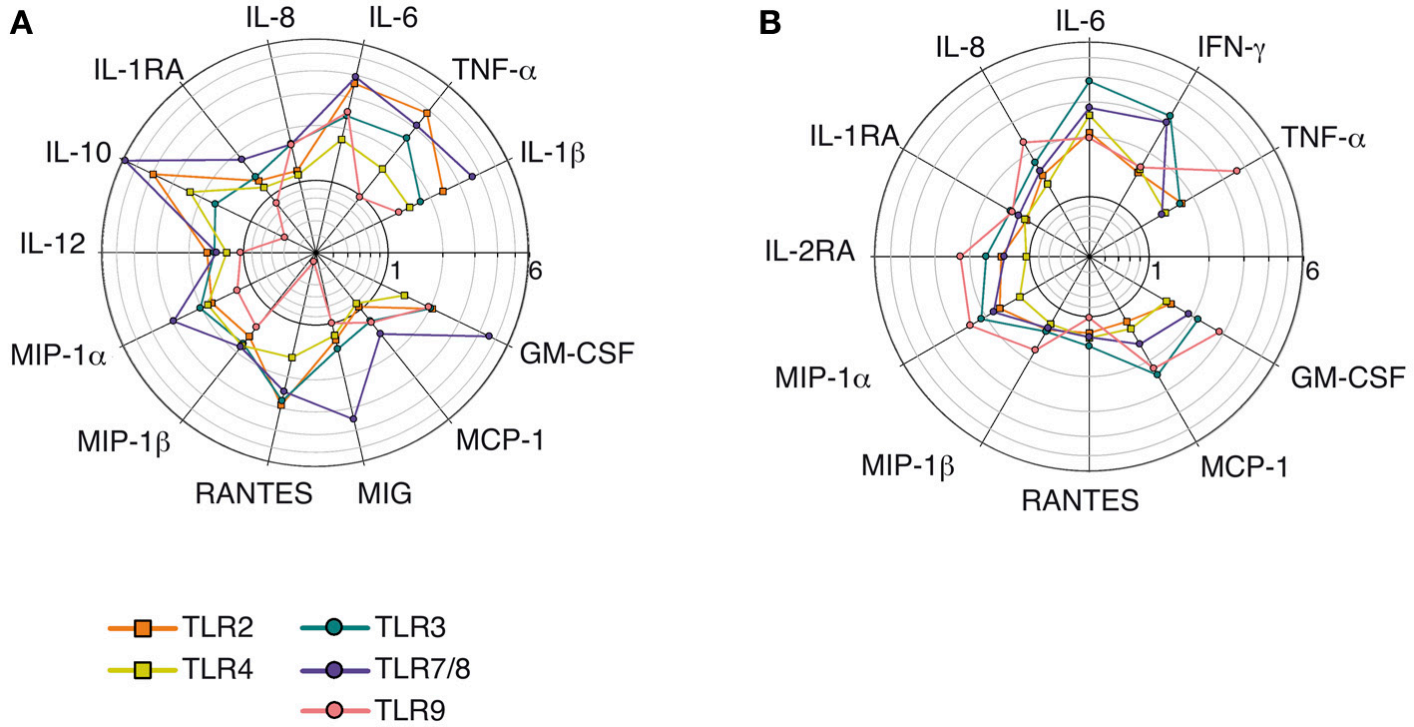
**Comparison of the cytokine profiles and median fold increases of cytokine secretion by dMs and dNK cells stimulated with TLR agonists**. The median fold increases in cytokine secretion after TLR2, TLR3, TLR4, TLR7/8, and TLR9 stimulations of dMs **(A)** and dNK cells **(B)** are summarized on the same polar plot.

Stimulated dMs and dNK cells display distinct cytokine profiles: IL-10, IL-1β, IL-12, and MIG upregulation being specific of dMs, whereas only dNK cells enhanced IFN-γ and IL-2RA secretion (Figure 5).

No major differences were seen between dMs stimulated by surface TLRs sensing microbial membrane patterns and intracellular TLRs sensing microbial nucleic acids (Figure 5A), although only intracellular TLR3, TLR7/8, and TLR9 activation significantly enhanced MCP-1 and IL-8 secretion. In addition, TLR9 activation of dMs resulted in an intriguing cytokine profile (Figure 5A): this was the only condition in which secretion of the chemokine RANTES and the anti-inflammatory cytokines IL-1RA and IL-10 was not enhanced or was inhibited.

IL-1RA, MCP-1, and GM-CSF secretion by dNK cells was enhanced more strongly by intracellular TLRs that sense microbial nucleic acid (TLR3, TLR7/8, and TLR9) than by activation of cell-surface TLRs that sense microbial membrane patterns (TLR2 and TLR4) (Figure 5B and Table 4). In addition, the strongest enhancement of IFN-γ secretion by dNK cells was observed after activation of the intracellular TLR3 and TLR7/8 (Figure 5B and Table 4).

## Discussion

DMs and dNK innate immune cells present in the decidua, have a crucial role in embryo implantation and fetal development (Hanna et al., 2006; Lash et al., 2006a,b; Houser et al., 2011). Their involvement in the initiation of immune responses to pathogens was strongly suspected but remained to be demonstrated.

We report for the first time that, during the first trimester of pregnancy, human decidual macrophages and NK cells (i) differentially express TLR1-9 mRNAs, (ii) express TLR2, TLR3, and TLR4 proteins, and (iii) possess functional TLRs that are known to sense a broad variety of PAMPs, including microbial membrane and nucleic acid patterns. Specific agonists of TLR2, TLR3, TLR4, TLR7/8, and TLR9 induced dMs and dNK cells to increase their secretion of pro-inflammatory and anti-inflammatory cytokines, as well as cytokines and chemokines involved in immune cell crosstalk. DMs and dNK cells displayed different cytokine profiles: IL-1β, IL-10, and IL-12 secretion was specific to dM cells, whereas IFN-γ secretion was specific to dNK cells. Most of the cytokines secreted by both cell types were present at higher concentrations in dM supernatants.

An appropriate pro-inflammatory/anti-inflammatory cytokine balance within the decidua basalis is required to support fetal development, and must therefore be preserved during antimicrobial responses triggered by TLR activation, in order to limit inflammation-induced tissue damage and thus to maintain a successful pregnancy.

Besides their pro-inflammatory actions, IL-6 induces secretion of the angiogenic factor VEGF, while IL-8 is involved in fetal implantation by stimulating placental trophoblast cell invasion within the decidua (Hanna et al., 2006; Barrientos et al., 2009). Thus, these cytokine have a crucial builder function during pregnancy (Le Bouteiller and Tabiasco, 2006). IL-6 and IL-8 were secreted by both dMs and dNK cells, and their secretion was enhanced by TLR activation. Thus, dMs and dNK cells probably maintain their IL-6 and IL-8-related builder function even after TLR activation.

The changes observed here in the dM cytokine profile after TLR activation are compatible with the results of previous transcriptome studies suggesting that dMs present features of both pro-inflammatory and tolerogenic macrophages (Houser et al., 2011; Svensson et al., 2011). Indeed, dMs increased IL-1β and TNF-α secretion (pro-inflammatory cytokines), together with IL-6 and IL-8, in response to TLR activation, while secretion of the anti-inflammatory cytokines IL-1RA and IL-10 was also enhanced. IL-1β secretion is tightly regulated and requires the activation of inflammasome complex containing activated caspase 1 for the cleavage of cytosolic inactive pro-IL-1β protein in mature IL-1β, which is secreted (Martinon et al., 2002; Dinarello, 2009). Whereas TLRs activation is known to induce pro-IL-1β protein expression, inflammasome complex activation involves the recognition of pathogen or damage-cell associated molecular patterns by intracellular inflammasome sensor (Dinarello, 2009). However, constitutively activated inflammasome were reported in human blood monocytes and dendritic cells (Nguyen et al., 2007; Netea et al., 2009; Carta et al., 2011; He et al., 2013; Snodgrass et al., 2013) and suggest that the regulation of inflammasome-mediated IL-1β production in APCs is cell-specific. The secretion of IL-1β by dMs in the absence of exogeneous TLR activation suggests that dMs could have an activated inflammasome in the decidual environment.

Once secreted, IL-1β pro-inflammatory activity is regulated by its antagonist IL-1RA. IL-1RA competes with IL-1β for its receptors and has an important role in regulating inflammation (Arend, 2002). We found that the IL1RA/IL-1β balance in dM supernatants in both basal and TLR-stimulated conditions always favored IL-1RA. IL-1RA secretion by dNK cells was also enhanced by TLR stimulation. IL-1RA secretion by dMs and dNK cells after TLR activation might help to counterbalance the pro-inflammatory action of IL-1β and to maintain a fetotolerant microenvironment. IL-10 plays a crucial role in tissue homeostasis, by preventing bystander tissue damage during inflammatory processes such as inflammatory bowel disease (Paul et al., 2012). Interestingly, mice lacking IL-10 expression are more susceptible to preterm delivery when challenged with TLR4 or TLR9 ligands (Robertson et al., 2007; Thaxton et al., 2009). In this latter mouse model, IL-10 was shown to prevent fetal loss by inhibiting placental invasion by cytotoxic NK cells and macrophages and by down-regulating pro-inflammatory cytokines, including TNF-α (Murphy et al., 2009; Thaxton et al., 2009). IL-10 has also been reported to modulate the responses of primary human placental cells to TLR2 and TLR4 ligands, reducing their secretion of IL-1β, IL-6, and IL-8 (Bayraktar et al., 2009). Since dMs spontaneously secrete IL-10 and TLR2, TLR3, TLR4, and TLR7/8 activation induces a strong secretion of IL-10, a cross-talk between dMs and trophoblast placental cells might occur. The peculiar cytokine profile of dMs observed under TLR activation with the secretion of both IL-1β, TNF-α, IL-6 pro-inflammatory and IL-10, IL-1RA anti-inflammatory cytokines as well as a low IL-12 secretion, is closely related to the cytokine profile of the immunoregulatory Macrophage M2b subset (Mantovani et al., 2004). Enhanced IL-10 secretion by dMs after TLR activation might help maintain fetal tolerance by damping the action of pro-inflammatory cytokines, thus underlining the tolerogenic function of dMs.

Our results reveal that TLR4 activation induced the lowest cytokine secretion by dMs. TLR4 activation of monocyte derived macrophages (MDM) in the same experimental conditions gave very high fold increase of TNF-α, IL-6, IL-12, IL-10, and β-chemokines (data not shown). Peripheral blood monocytes were shown to display a lower TLR4-induced cytokine secretion when they were pre-exposed to trophoblast placental cells (Fest et al., 2007). This education of monocytes by trophoblasts could explain why TLR4-induced cytokine secretion of *ex vivo* primary dMs which are in close contact with placental cells is low.

Interestingly, the dM cytokine profile observed after TLR9 activation was distinct from that induced by the other TLR agonists. Indeed, IL-1RA and IL-10 secretion by TLR9-activated dMs was not enhanced and was even sometimes reduced. Several observations suggest that pre-eclampsia (PE), a shallow implantation syndrome, could results from a pro/anti-inflammatory cytokine imbalance due to inappropriate TLR activation (Sado et al., 2011; Chatterjee et al., 2012). Emerging evidence suggests that TLR9 stimulation by mitochondrial DNA released from necrotic trophoblasts could induce local and systemic inflammatory responses and thereby contribute to the development of PE (Goulopoulou et al., 2012; Scharfe-Nugent et al., 2012). Moreover, a decrease in IL-10-expressing dMs has been observed in the decidua of PE patients (Schonkeren et al., 2011). Our finding that TLR9 activation enhances dMs secretion of pro-inflammatory cytokines but not that of the anti-inflammatory cytokines IL-10 and IL-1RA suggests a disruption of the pro/anti-inflammatory cytokine balance essential to maintain local materno-fetal tolerance.

Like their peripheral blood (pNK) and non-pregnancy uterine (uNK) counterparts, dNK cells exhibited enhanced IFN-γ secretion after TLR activation (Sivori et al., 2004; Eriksson et al., 2006; Kim et al., 2012; Souza-Fonseca-Guimaraes et al., 2012). Direct sensing by pNK cells of TLR3 and TLR9 agonists, as well as human cytomegalovirus (HCMV) and *Mycobacterium bovis* through TLR2, is reported to induce their IFN-γ-producer effector function and to increase their cytolytic activity (Sivori et al., 2004; Marcenaro et al., 2008; Muntasell et al., 2013). Interestingly, a recent study shows that dNK cells, which produce cytokines and chemokines during normal pregnancy, develop cytotoxic activity against HCMV-infected autologous decidual fibroblasts (Siewiera et al., 2013). Our results show that activation of dNK cells through their TLRs can activate their IFN-γ-secreting effector function. Whether or not TLR activation also shapes the cytotoxic effector function of dNK cells remains to be determined. DMs are not detected in purified dNK cell samples, however it cannot be totally excluded that remaining stromal decidual cells could indirectly support dNK cell IFN-γ production (Schaefer et al., 2005; Patel et al., 2012).

TLR activation of dMs and dNK cells enhances the secretion of several cytokines and chemokines involved in immune cell crosstalk. Among them, GM-CSF has a key role in embryo implantation and development, as it acts as a growth factor for trophoblastic cells (Robertson, 2007). Indeed, GM-CSF injection is reported to prevent fetal loss in an abortion-prone mouse model (Chaouat et al., 1990). Moreover, GM-CSF secretion by activated dNK cells was recently shown to enhance primary trophoblast migration, thus favoring placentation (Xiong et al., 2013). Beside its role in placentation, GM-CSF is involved in monocyte/macrophage recruitment, while MCP-1 also recruits NK cells (Hamilton, 2002; Robertson, 2002; Deshmane et al., 2009). The β-chemokines MIP-1α, MIP-1β, and RANTES recruit CCR5-expressing cells (monocytes, NK, and T cells); monocyte and CD56^bright^ pNK cell recruitment through MIP-1α secretion by placental cells has also been reported (Drake et al., 2001). MCP-1 and β-chemokines are reported to enhance pNK cytolytic activity (Taub et al., 1995; Robertson, 2002). Here we show that secretion of these chemokines by both dMs and dNK cells is enhanced by TLR activation. Together, these chemokines might enable the recruitment of a large panel of immune cells able to mount an appropriate response against invading pathogens, while GM-CSF promotes trophoblast invasion and maintains placentation.

The TLR-induced cytokine profile of dMs and dNK cells could influence the T CD4^+^ cell responses. The TLR activation of dNK cells results in the enhancement of IFN-γ, which was shown to prevent Th2 CD4^+^ T cells proliferation and cytokine synthesis (Oriss et al., 1997). dMs secrete IL-10 cytokine known to inhibit proliferation and cytokine response of both Th1 and Th2 CD4^+^ T cells (de Waal Malefyt et al., 1991; Fiorentino et al., 1991; Ding and Shevach, 1992; Del Prete et al., 1993; Joss et al., 2000). Thus, TLR-induced IL-10 secretion by dMs may prevent the over proliferation and over activation of CD4^+^ T cells.

Successful pregnancy was associated with a shift toward a Th2 cytokine biais, whereas a shift toward Th1 cytokine was associated with recurrent spontaneous abortions (Raghupathy, 2001). Nevertheless, a moderate Th1 inflammatory environment controlled by decidual regulatory T cells (Treg) is required during early pregnancy (Mjosberg et al., 2010) and a decrease of decidual Treg cells was associated with miscarriage (Sasaki et al., 2004; Inada et al., 2013). A cross-talk between dMs and dNK cells was shown to be involved in Treg induction through the secretion of IFN-γ by dNK cells, which allows IDO secretion by dMs, which in turn induces Treg (Vacca et al., 2010). Moreover, decidual Treg were shown to inhibits autologous T cells proliferation and to reduce both fetus-specific and non-specific immune responses (Tilburgs et al., 2008). Under TLR activation, the enhancement of IFN-γ secretion by dNK cells is thus compatible with fetal tolerance together with the defense against pathogens.

Interestingly, dMs secrete IL-15 as well as IL-12, secretion of which is increased after TLR activation. NK cell priming by IL-15 is known to be required for downstream NK cell activation (Lucas et al., 2007) and IL-15 is a growth/activation factor for dNK cells (Verma et al., 2000). As IL-15 is expressed within the decidua (Marlin et al., 2011), dNK cells are physiologically primed. Our results suggest that dMs may be involved in this mechanism. IL-12 is reported to potentiate IFN-γ production by pNK cells (Fehniger et al., 1999). IFN-γ release by TLR7/8-activated pNK cells, and by *Trypanozoma cruzi*-activated cord blood NK cells through TLR2/4, is dependent on IL-12 production by accessory cells (Hart et al., 2005; Guilmot et al., 2013). Moreover, pNK cell cytotoxic activity induced through TLR7/8 or TLR3 activation is enhanced by an IL-12-related mechanism (Hart et al., 2005). Thus, IL-15 secretion by dMs and the increase in IL-12 production after TLR activation could respectively prime dNK cells and enhance their IFN-γ secretion in the presence of a pathogen.

In conclusion, we report here for the first time that decidual macrophages and natural killer cells express functional TLR2, TLR3, TLR4, TLR7/8, and TLR9, and are thus able to sense a large panel of PAMPs. The cytokine profiles induced by TLR activation are compatible with simultaneous initiation of antimicrobial responses, control of the inflammatory response, and maintenance of the fetotolerant immune environment.

## Author contributions

Conceived and designed the experiments: Marion Duriez, Héloïse Quillay, Romain Marlin, Françoise Barré-Sinoussi, Marie-Thérèse Nugeyre, Elisabeth Menu. Performed the experiments: Marion Duriez, Héloïse Quillay, Hicham El Costa, Claude Cannou, Romain Marlin, Marie-Thérèse Nugeyre. Analyzed the data: Marion Duriez, Héloïse Quillay, Yoann Madec, Marie-Thérèse Nugeyre, Elisabeth Menu. Contributed reagents/materials/analysis tools: Claire de Truchis, Mona Rahmati. Wrote the paper: Marion Duriez, Héloïse Quillay, Yoann Madec, Hicham El Costa, Romain Marlin, Marie-Thérèse Nugeyre, Elisabeth Menu.

### Conflict of interest statement

The authors declare that the research was conducted in the absence of any commercial or financial relationships that could be construed as a potential conflict of interest.
